# Efficacy of Platelet-Rich Plasma (PRP) in the Management of Rotator Cuff Tears: A Case Series From a Tertiary Care Center

**DOI:** 10.7759/cureus.88965

**Published:** 2025-07-29

**Authors:** Muhammad Awais Iqbal, Muhammad Mannan, Sarmad Khalil, Malik Haroon Hameed, Faisal Karim, Zawar Ahmad, Daniel Carter, Nayan Shrivastava

**Affiliations:** 1 Trauma and Orthopedics, Ghurki Trust Teaching Hospital, Lahore, PAK; 2 Trauma and Orthopedics, The Dudley Group NHS Foundation Trust, Dudley, GBR; 3 Orthopedic Surgery, University Hospitals Birmingham NHS Foundation Trust, Birmingham, GBR; 4 Trauma and Orthopedics, University Hospitals Birmingham NHS Foundation Trust, Birmingham, GBR; 5 Trauma and Orthopedics, Kettering General Hospital NHS Foundation Trust, Kettering, GBR; 6 Orthopedics, Sancheti Institute for Orthopedics and Rehabilitation, Pune, IND

**Keywords:** functional recovery, healing rate, platelet rich plasma, rotator cuff tear, ultrasound assessment

## Abstract

Objective: To evaluate the short-term tendon healing response to a single ultrasound-guided autologous platelet-rich plasma (PRP) injection in patients aged 25-60 years with clinically and ultrasonographically confirmed unilateral, partial- to full-thickness rotator cuff tears.

Materials and methods: This descriptive case series was conducted at the Department of Orthopedic and Spine Surgery, Ghurki Trust Teaching Hospital, Lahore, from April 25 to October 24, 2018. A total of 90 patients aged 25 to 60 years, with clinically and ultrasonographically confirmed partial- and full-thickness rotator cuff tears, were enrolled. Patients with diabetes mellitus, bone infections (e.g., osteomyelitis), or muscular and skeletal dystrophies were excluded. All patients received a single PRP injection administered by the principal investigator. Follow-up assessments were conducted over a 12-week period, and tendon healing was evaluated using ultrasonography. Complete healing was defined as the absence of a tear on follow-up imaging.

Results: Ninety patients were included, mean age: 41.46 ± 9.05 years; 61 males (67.78%) and 29 females (32.22%). Most patients (55.56%) were aged 41-60 years, and right shoulder involvement was observed in 56.67% of cases. At 12 weeks, complete tendon healing was noted in 62 patients (68.89%), while 28 patients (31.11%) showed no healing. Stratified analysis revealed no statistically significant association between healing outcomes and age (p = 0.839), gender (p = 0.141), symptom duration (p = 0.900), BMI (p = 0.504), or affected side (p = 0.327).

Conclusion: PRP therapy was associated with a high rate of tendon healing on ultrasonographic assessment at 12 weeks in patients with partial- to full-thickness rotator cuff tears. While these findings suggest potential short-term structural benefits, they should be interpreted with caution due to the study’s descriptive design, lack of a control group, and reliance on ultrasound. Although PRP may be a useful adjunct in conservative management, especially for non-surgical candidates, current evidence supports its role primarily in symptom relief. Larger randomized controlled trials with standardized protocols and functional outcomes are needed to establish its long-term efficacy.

## Introduction

The shoulder is one of the most mobile joints in the human body and, consequently, a frequent source of dynamic instability and functional impairment. This instability often results from a spectrum of pathologies, including rotator cuff tears, subacromial and subcoracoid impingement, calcific tendonitis, and osteoarthritis. Such conditions are not only common but are also associated with a progressive decline in upper limb function, particularly among older adults [1].

Rotator cuff disorders in particular have a complex and multifactorial etiology, often leading to gradual tendon degeneration over time [2]. Traumatic rotator cuff tears tend to occur more frequently in younger individuals, especially following high-impact injuries such as falls or during overhead athletic activities. In contrast, degenerative tears are more prevalent among the aging population, correlating with chronic overuse and intrinsic tendon degeneration. Full-thickness rotator cuff tears are reported in approximately 5% to 40% of individuals, depending on the population studied, and their prevalence increases markedly with age [3]. In a classic cadaveric study, Bigliani et al. found that up to 39% of individuals over the age of 60 exhibited full-thickness rotator cuff tears, with an even higher incidence of partial-thickness lesions [4,5].

Accurate diagnosis of rotator cuff pathology is essential for guiding appropriate treatment. Advanced imaging modalities such as ultrasonography (USG), magnetic resonance imaging (MRI), and magnetic resonance arthrography (MR arthrography) offer valuable insights into the structural integrity of the rotator cuff and surrounding tissues [6]. A meta-analysis published in 2009, using surgical findings as the reference standard, reported that MRI had a sensitivity and specificity of 92.1% and 92.9%, respectively, for detecting full-thickness tears [7]. While MRI remains the gold standard in many settings, its cost, limited availability, and contraindications in certain patient populations can pose practical challenges, particularly in resource-constrained environments [8].

Conservative (non-operative) management continues to be the first-line approach for many patients with rotator cuff tears, particularly those with partial-thickness tears or individuals unfit for surgery. Such management typically includes physical therapy, lifestyle modification, and pharmacological interventions such as non-steroidal anti-inflammatory drugs (NSAIDs), analgesics, and corticosteroid injections [9]. In recent years, there has been growing interest in orthobiologics, particularly platelet-rich plasma (PRP), as a minimally invasive treatment modality with the potential to enhance tendon healing and reduce pain [10]. Although the biological rationale for PRP is well established, given its high concentration of growth factors such as platelet-derived growth factor (PDGF), transforming growth factor-beta (TGF-β), and vascular endothelial growth factor (VEGF), its clinical effectiveness remains a subject of debate, largely due to variability in preparation methods, injection techniques, and patient selection [11].

Evidence from a previous study suggested that 67% of patients receiving PRP as an adjunct to surgical rotator cuff repair demonstrated complete tendon-to-bone healing at 12 weeks, indicating a promising therapeutic potential [12]. However, despite international data, local evidence evaluating the use of PRP in nonoperatively managed rotator cuff tears is currently lacking. Given this gap in the literature, the present study was undertaken to evaluate the clinical outcomes of PRP therapy in patients with rotator cuff tears at a tertiary care center in Pakistan. The goal was to generate context-specific data that may guide future clinical decision-making and facilitate the integration of PRP into conservative treatment protocols in similar healthcare settings.

## Materials and methods

Study design

This study was a prospective, descriptive case series conducted at the Department of Orthopedic and Spine Surgery, Ghurki Trust Teaching Hospital, Lahore, over a six-month period from April 25, 2018, to October 24, 2018. The College of Physicians and Surgeons, Pakistan, issued approval CPSP/REU/OSG-2016-080-1677. The objective was to evaluate the short-term tendon healing response to a single ultrasound-guided autologous PRP injection in patients aged 25-60 years with clinically and ultrasonographically confirmed unilateral, small- to medium-sized, partial- to full-thickness rotator cuff tears.

A rotator cuff tear was defined as a pathological lesion involving one or more tendons of the four rotator cuff muscles (supraspinatus, infraspinatus, subscapularis, or teres minor) confirmed through USG. Patients presenting with shoulder pain persisting for more than four weeks and imaging evidence of a tear were considered eligible. Complete healing was defined as the absence of any residual tear on follow-up USG by a musculoskeletal (MSK) radiologist at 12 weeks, with reconstitution of tendon continuity and restoration to its normal anatomical position. 

Inclusion and exclusion criteria

The sample size was calculated using the World Health Organization (WHO) sample size calculator, based on a 95% confidence level, an expected healing rate of 67%, and a 10% margin of error. A total of 90 patients were enrolled using non-probability, consecutive sampling (Table 1).

**Table 1:** Inclusion and exclusion criteria

Inclusion criteria	Exclusion criteria
Age between 25 and 60 years	Known diabetes mellitus (random blood sugar > 200 mg/dL at screening)
Either gender	Bilateral rotator cuff pathology confirmed on clinical or imaging evaluation
Tear resulting from either a traumatic event or a degenerative onset	Active or prior bone infections (e.g., osteomyelitis), as confirmed by radiographic evidence
Clinically diagnosed shoulder pain persisting for more than four weeks	Documented muscular or skeletal dystrophies
Ultrasonographic confirmation of partial to full-thickness tear	Massive or retracted tears involving multiple tendons
Tear primarily involving the supraspinatus tendon	Prior shoulder surgery or corticosteroid injection within the past six months
Small to medium-sized tears suitable for conservative management	

Data collection

Eligible patients were recruited from the outpatient department of the Orthopedic and Spine Surgery Unit after providing written informed consent. Baseline demographic and clinical data, including age, sex, body mass index (BMI), symptom duration, and affected shoulder side, were documented using a structured proforma.

Each patient received a single dose of autologous PRP, prepared from 20 mL of autologous venous blood. The PRP was processed using a double spin centrifugation technique with a commercially available PRP preparation kit, yielding approximately 4-5 mL of PRP with a platelet concentration four to five times higher than baseline. The final product was classified as leukocyte-rich PRP, as confirmed by cell count analysis. PRP was prepared under sterile conditions and administered by the principal investigator into the affected rotator cuff tendon under ultrasound guidance.

During the 12-week follow-up period, patients were permitted to use NSAIDs for pain relief. No additional intra-articular injections, corticosteroids, or structured physiotherapy protocols were allowed to minimize treatment variability. Follow-up assessments were conducted in the outpatient setting, and tendon healing was evaluated by an MSK radiologist using USG. Complete healing was defined as the absence of any residual tear on ultrasound and restoration of tendon continuity. Patients who did not exhibit complete healing, defined as the absence of a residual tear on USG and restoration of tendon continuity, were subsequently managed according to institutional clinical protocols, which included further imaging (e.g., MRI) and consideration for surgical intervention if symptoms persisted.

Statistical analysis

All data were entered and analyzed using IBM SPSS Statistics for Windows, Version 24 (Released 2016; IBM Corp., Armonk, New York, United States). Continuous variables such as age, BMI, and duration of symptoms were expressed as mean ± standard deviation. Categorical variables, including gender, anatomical site of the tear, and healing status, were presented as frequencies and percentages.

To assess associations between complete healing and patient characteristics, stratification was performed by age, gender, BMI, symptom duration, and side of involvement. The chi-square test was applied to evaluate statistical significance, with a p-value ≤ 0.05 considered indicative of a statistically significant association.

## Results

The age range in this study was from 25 to 60 years, with a mean age of 41.46 ± 9.05 years. The majority of the patients, 50 (55.56%), were between 41 and 60 years of age. Out of these 90 patients, 61 (67.78%) were male and 29 (32.22%) were female, with a male-to-female ratio of 2.1:1. The mean duration of symptoms is 11.02 ± 5.54 weeks. The mean BMI is 29.30 ± 1.95 kg/m². Out of the 90 patients, complete healing after 12 weeks of PRP therapy was observed in 62 patients (68.89%), while 28 (31.11%) did not achieve complete healing (Table 2).

**Table 2:** Demographic and clinical profile of patients (n = 90) No statistical test was applied for this table as it describes baseline characteristics.

Variable	Category	n (%)	Mean ± SD
Age (years)	25–40	40 (44.44%)	
	41–60	50 (55.56%)	41.46 ± 9.05
Gender	Male	61 (67.78%)	
	Female	29 (32.22%)	
Duration of Symptoms	5–12 weeks	57 (63.33%)	
	>12 weeks	33 (36.67%)	11.02 ± 5.54 weeks
BMI (kg/m²)	≤30	56 (62.22%)	
	>30	34 (37.78%)	29.30 ± 1.95
Anatomical Site	Right Shoulder	51 (56.67%)	
	Left Shoulder	39 (43.33%)	
Outcome	Complete Healing	62 (68.89%)	
	No Healing	28 (31.11%)	

Among patients aged 25-40 years, 28 out of 40 (70.0%) achieved complete healing, while in the 41-60 age group, 34 out of 50 (68.0%) healed (p = 0.839). Among males, 39 out of 61 (63.9%) achieved complete healing, compared to 23 out of 29 (79.3%) females (p = 0.141). For the duration of symptoms, 39 out of 57 (68.4%) with a five- to 12-week duration healed completely, versus 23 out of 33 (69.7%) with symptoms >12 weeks (p = 0.900). Patients with BMI ≤ 30 had 40 out of 56 (71.4%) complete healing, while 22 out of 34 (64.7%) patients with BMI > 30 healed (p = 0.504). Lastly, 33 out of 51 (64.7%) with right shoulder involvement healed completely versus 29 out of 39 (74.4%) with left shoulder involvement (p = 0.327) (Table 3).

**Table 3:** Stratification of healing outcomes by patient characteristics Statistical test used: chi-square test, p-value ≤ 0.05 considered significant.

Variable	Category	Complete healing n (%)	No healing n (%)	Chi-square value	p-value
Age (years)	25–40	28 (70.0%)	12 (30.0%)	0.041	0.839
	41–60	34 (68.0%)	16 (32.0%)		
Gender	Male	39 (63.9%)	22 (36.1%)	2.174	0.141
	Female	23 (79.3%)	6 (20.7%)		
Duration of Symptoms	5–12 weeks	39 (68.4%)	18 (31.6%)	0.016	0.900
	>12 weeks	23 (69.7%)	10 (30.3%)		
BMI (kg/m²)	≤30	40 (71.4%)	16 (28.6%)	0.446	0.504
	>30	22 (64.7%)	12 (35.3%)		
Anatomical Site	Right Shoulder	33 (64.7%)	18 (35.3%)	0.964	0.327
	Left Shoulder	29 (74.4%)	10 (25.6%)		

## Discussion

PRP delivers a concentrated source of alpha-granules containing key growth factors such as TGF-β and VEGF, which contribute to soft tissue regeneration through enhanced collagen production and cellular proliferation [13,14]. By promoting angiogenesis and recruiting mesenchymal stem cells to the site of injury, PRP provides an environment conducive to tissue healing and remodeling. It has demonstrated safety and therapeutic efficacy across a range of disciplines, including orthopedic surgery, dentistry, dermatology, and aesthetic medicine. In orthopedics, PRP has gained considerable interest for managing MSK injuries and osteoarthritis due to its minimally invasive nature and low complication profile. Studies involving intra-articular PRP injections in patients with ankle osteoarthritis have shown notable improvements in both pain reduction and functional scores, without major adverse events [15,16].

The present study adds to the growing evidence supporting PRP as a treatment modality for rotator cuff tears. Among 90 patients treated with a single PRP injection and followed for 12 weeks, 62 (68.89%) demonstrated complete healing on USG. The remaining 28 patients (31.11%) who did not achieve complete healing were managed according to institutional clinical protocols, which included extended conservative treatment, repeat imaging (such as MRI), or referral for surgical intervention based on persistent symptoms and functional limitation. These findings suggest that PRP may offer substantial short-term benefits in tendon healing for appropriately selected patients, potentially reducing the need for surgical intervention in a significant proportion of cases. Godek et al. similarly reported improved pain scores and faster rehabilitation when PRP was used as an adjunct in rotator cuff repair, with a notable reduction in re-tear rates and quicker return to daily activities [17].

Randelli et al. also reported improved pain and functional outcomes in patients receiving PRP during arthroscopic rotator cuff repair, as evidenced by an increase in Constant Murley Scores at 12 weeks postoperatively, with no reported adverse effects [9,12]. However, it is essential to acknowledge that inconsistencies in PRP outcomes may stem from heterogeneity in its preparation, concentration, activation method, and injection protocols. These variations complicate cross-study comparisons and likely contribute to the lack of consensus regarding its overall efficacy.

To address this, some researchers have proposed the use of allogeneic PRP, which offers several potential advantages over autologous formulations. Single-donor PRP products obtained from established blood bank procedures are safer, more cost-effective, and more consistent in composition. Such preparations standardize key variables such as platelet count, leukocyte concentration, centrifugation force, and processing time, thus improving reproducibility across clinical trials [18]. In addition, platelet apheresis reduces spontaneous platelet activation compared to manual techniques, potentially enhancing therapeutic efficacy [19].

Despite promising results, not all trials have demonstrated consistent benefits. Kesikburun et al. found no significant advantage of PRP over conventional conservative therapy in patients with chronic rotator cuff tendinopathy, though the injection site (subacromial space rather than intratendinous) may have influenced outcomes [10]. Similarly, Pang et al. observed clinical benefits with both intratendinous PRP and corticosteroid injections, raising questions about relative efficacy [20].

A 12-month follow-up study reported a 15% improvement in pain and function following PRP, along with a reduced need for surgical intervention. However, this must be interpreted cautiously in light of evidence suggesting poorer long-term outcomes in patients treated with repeated corticosteroid injections.

Castricini et al. compared standard arthroscopic double-row repair with and without PRP augmentation in patients with small- to medium-sized rotator cuff tears. At 16 months, no significant difference in Constant-Murley Scores or MRI-based tendon integrity was observed, although a lower re-tear rate in the PRP group (2.5%) compared to controls (10.5%) approached statistical significance (P = 0.07) [21,20].

Malavolta et al. similarly found no significant differences in clinical or radiographic outcomes when PRP was added to single-row repairs of small full-thickness supraspinatus tears, except for a marginal improvement in University of California at Los Angeles (UCLA) scores at 12 months (P = .046), which became non-significant after correction for multiple comparisons [22].

Weber et al. further corroborated these findings in a double-blinded trial, reporting no statistically significant improvement in clinical outcomes or re-tear rates at 12 months postoperatively between patients receiving PRP and controls [23].

In the orthopedics field, PRP has shown efficacy in accelerating bone healing, particularly in cases of delayed union, and in managing chronic tendinopathies such as lateral epicondylitis and patellar tendinopathy [24]. Its anti-inflammatory and regenerative properties also extend to dental, dermatologic, and cosmetic applications, where it is used to enhance skin texture, reduce wrinkles, and manage alopecia and scars [25,26].

Limitations

This study has several limitations. Firstly, the single-center design and relatively small sample size limit the generalizability of the findings. The use of non-probability, consecutive sampling introduces the possibility of selection bias, potentially skewing the observed treatment effects. Secondly, the absence of a control group prevents direct assessment of PRP’s efficacy relative to standard conservative treatments, such as physical therapy or corticosteroid injections.

Thirdly, while USG is a useful and cost-effective imaging modality, it is operator-dependent and may lack the sensitivity and specificity of MRI for detecting subtle tendon pathology or partial tears. The short 12-week follow-up period, although sufficient for assessing early healing, may not reflect the long-term structural and functional outcomes or the durability of tendon integrity.

Future studies with larger, multicenter cohorts, longer follow-up durations, and inclusion of validated clinical outcome measures (e.g., Constant Score, American Shoulder and Elbow Surgeons Score (ASES), Visual Analog Scale (VAS)) are needed to more comprehensively assess the role of PRP in rotator cuff tear management. Randomized controlled trials with standardized PRP protocols are particularly essential to clarify the true efficacy and reproducibility of this therapeutic approach.

## Conclusions

This study found that PRP therapy was associated with a high rate of tendon healing on ultrasonographic assessment at 12 weeks in patients with partial- to full-thickness rotator cuff tears. While these findings suggest potential short-term structural benefits, it is important to interpret them cautiously given the descriptive nature of the study, absence of a control group, and reliance on USG alone for outcome evaluation.

Given PRP’s minimally invasive nature, low complication profile, and ease of outpatient administration, it may represent a promising adjunct in the conservative management of rotator cuff pathology, particularly for patients unfit for surgery or preferring nonoperative care. However, current evidence from well-designed clinical trials largely supports PRP’s benefit in symptom improvement rather than structural healing.

Therefore, while our study contributes valuable local data, definitive conclusions regarding PRP’s superiority in tendon healing cannot be drawn. Further randomized controlled trials with standardized PRP protocols, longer follow-up, and functional outcome measures are warranted to clarify its clinical role and long-term efficacy.
